# Fibroblasts direct differentiation of human breast epithelial progenitors

**DOI:** 10.1186/s13058-020-01344-0

**Published:** 2020-09-29

**Authors:** Mikkel Morsing, Jiyoung Kim, René Villadsen, Nadine Goldhammer, Abbas Jafari, Moustapha Kassem, Ole William Petersen, Lone Rønnov-Jessen

**Affiliations:** 1 Department of Cellular and Molecular Medicine, University of Copenhagen, Copenhagen, Denmark; 2 Danish Stem Cell Centre, University of Copenhagen, Copenhagen, Denmark; 3 Present Address: Division of Translational Cancer Research, Department of Laboratory Medicine, Lund University, Lund, Sweden; 4 Laboratory of Molecular Endocrinology, KMEB, Department of Endocrinology, Odense University Hospital and University of Southern Denmark, Odense, Denmark; 5 Section for Cell Biology and Physiology, Department of Biology, University of Copenhagen, Copenhagen, Denmark

**Keywords:** Breast, Fibroblast, Epithelial progenitors, Differentiation

## Abstract

**Background:**

Breast cancer arises within specific regions in the human breast referred to as the terminal duct lobular units (TDLUs). These are relatively dynamic structures characterized by sex hormone driven cyclic epithelial turnover. TDLUs consist of unique parenchymal entities embedded within a fibroblast-rich lobular stroma. Here, we established and characterized a new human breast lobular fibroblast cell line against its interlobular counterpart with a view to assessing the role of region-specific stromal cues in the control of TDLU dynamics.

**Methods:**

Primary lobular and interlobular fibroblasts were transduced to express human telomerase reverse transcriptase (hTERT). Differentiation of the established cell lines along lobular and interlobular pathways was determined by immunocytochemical staining and genome-wide RNA sequencing. Their functional properties were further characterized by analysis of mesenchymal stem cell (MSC) differentiation repertoire in culture and in vivo. The cells’ physiological relevance for parenchymal differentiation was examined in heterotypic co-culture with fluorescence-activated cell sorting (FACS)-purified normal breast primary luminal or myoepithelial progenitors. The co-cultures were immunostained for quantitative assessment of epithelial branching morphogenesis, polarization, growth, and luminal epithelial maturation. In extension, myoepithelial progenitors were tested for luminal differentiation capacity in culture and in mouse xenografts. To unravel the significance of transforming growth factor-beta (TGF-β)-mediated crosstalk in TDLU-like morphogenesis and differentiation, fibroblasts were incubated with the TGF-β signaling inhibitor, SB431542, prior to heterotypic co-culture with luminal cells.

**Results:**

hTERT immortalized fibroblast cell lines retained critical phenotypic traits in culture and linked to primary fibroblasts. Cell culture assays and transplantation to mice showed that the origin of fibroblasts determines TDLU-like and ductal-like differentiation of epithelial progenitors. Whereas lobular fibroblasts supported a high level of branching morphogenesis by luminal cells, interlobular fibroblasts supported ductal-like myoepithelial characteristics. TDLU-like morphogenesis, at least in part, relied on intact TGF-β signaling.

**Conclusions:**

The significance of the most prominent cell type in normal breast stroma, the fibroblast, in directing epithelial differentiation is largely unknown. Through establishment of lobular and interlobular fibroblast cell lines, we here demonstrate that epithelial progenitors are submitted to stromal cues for site-specific differentiation. Our findings lend credence to considering stromal subtleties of crucial importance in the development of normal breast and, in turn, breast cancer.

## Background

There is an increasing appreciation that the generic term “fibroblast” is not simply synonymous with any spindle-shaped stromal cell type manufacturing an acellular interstitial collagenous tissue. In mice, for example, separate fibroblast lineages govern the papillary and the reticular layers of the dermis [1]. Functionally, these fibroblasts also carry out important different functions related to epidermal and subcutaneous homeostasis, respectively [1]. In human tissue, fibroblasts have attracted most attention in relation to tumor formation. Here, they are referred to as myofibroblasts or cancer-associated fibroblasts (CAFs) and at different times have been considered as either facilitating or inhibiting tumor progression and thus offering potential new avenues of therapeutic intervention [2]. Indeed, mesenchymal cues are considered sufficient to induce malignant transformation [3]. In the human breast, initial transformation is thought to take place in epithelial progenitors residing in so-called terminal duct lobular units (TDLUs [4];). The TDLU is the functional unit of the human breast and consists of a branching terminal duct ending in varying numbers of acinus-like ductules, all of which are embedded in loose connective tissue (reviewed in [5, 6]). The loose connective tissue is unique for the TDLUs, which drain into the interlobular ducts, which in turn are embedded in a more dense connective tissue (reviewed in [5]). For this reason, several efforts have been made to characterize lobular fibroblasts as a separate lineage with functional properties [7–9]. Recently, we provided unequivocal evidence for the existence of a CD105^high^ TDLU-resident lobular fibroblast with properties different from interlobular fibroblasts [10]. While the CD105^high^ lobular fibroblasts resemble mesenchymal stem cells (MSCs) both by phenotype and function, CD26^high^ interlobular cells remain fibroblast restricted [10].

The epithelial compartments of lobules and ducts also differ. Thus, in addition to the obvious morphological difference between the compartments, epithelial progenitors, which differ by cytokeratin expression [11], have been identified in both ducts and TDLUs [12, 13]. Apparently, this difference is pre-programmed in myoepithelial progenitors at the apex of the hierarchy and maintained upon differentiation after several generations in culture and in vivo [11]. In light of the existence of functionally distinct fibroblasts in human skin [14], it is tempting to speculate that myoepithelial differentiation programs, at least in part, are governed by neighboring stromal cells. With the aim of unraveling critical aspects of normal breast development, and, in turn, gain insight into how stromal diversity impinges on epithelium during cancer development, we resolved that access to established fibroblast cell lines would be necessary not least for the sake of reproducibility.

We here embarked with hTERT immortalization of prospectively isolated lobular CD105^high^ and interlobular CD26^high^ human breast fibroblasts. We established two different fibroblast cell lines and show that they specifically direct the differentiation of primary epithelial cell progenitors.

## Methods

### Tissue

Normal breast tissue was obtained from women undergoing reduction mammoplasty for cosmetic reasons. Information about donors is restricted to the donor’s age at the time of surgery. The tissue was donated with written consent by donors who received information before surgery at a clinic in the Greater Copenhagen area, Denmark. The Regional Scientific Ethical Committees (Region Hovedstaden, H-2-2011-052) and the Danish Data Protection Agency (2011-41-6722) reviewed and approved the use and storage of human material. Some of the donated tissue has been included in other studies. Procedures for orthotopic injection of human cells into the mouse mammary fat pad or under the skin was reviewed and approved by the Danish National Animal Experiment Inspectorate (2017-15-0201-01315 and 2017-15-0201-01210).

### Cell isolation and cell culture

An established protocol for preparation and isolation of stromal cells and epithelial organoids was applied and can be found elsewhere [15]. We used four sets of primary CD105^high^/CD26^low^ lobular and CD105^low^/CD26^high^ interlobular human breast fibroblastic cells (HBFCs) from four different biopsies obtained from donors at 19, 20, 23, and 29 years of age, which had been isolated previously [10]. These cell strains as well as the two hTERT immortalized fibroblast cell lines (iHBFCs, iHBFC^CD105^ and iHBFC^CD26^, respectively), derived from a donor of the age of 20 years were maintained in DMEM/F-12 (DMEM:Ham’s F12 Nutrient Mixture (F12), 1:1 v/v, Life Technologies) supplemented with 5% fetal bovine serum (FBS, Sigma), 2 mM glutamine, and penicillin-streptomycin antibiotics (DMEM/F12-5%). The cultures were plated at a density of 5600 cells/cm^2^ in collagen coated flasks (Nunc, 8 μg collagen/cm^2^, PureColl, Cell Systems).

An hTERT immortalized MSC line, hMSC-TERT4 [16], referred to here as hMSC-TERT was cultured on plastic (Nunc) in Minimum Essential Medium (MEM, containing Earle’s salts and L-Glutamine, Gibco) supplemented with 10% FBS (South American Origin, Gibco) and 1% penicillin-streptomycin (Gibco) (MEM-10%) and split 1:4 at ~ 80% confluence. All cell cultures were maintained at 37 °C in a humidified atmosphere with 5% CO_2_ with medium change three times a week.

Population doubling level (PDL) was calculated as follows: PDL = 3.32 (log *I* − log *Y*) + X, where *I* is the cell number of the inoculum, *Y* is the cell yield, and *X* is the population doubling of the inoculum. The hTERT immortalized breast fibroblasts have currently been propagated for more than 80 passages (available through Ximbio, UK, IAHF, cat. no. 153783 and IEHF, cat. no. 153784).

### Viral transduction

Viral constructs used included human telomerase (pBabe-neo-hTERT, Addgene #1774, a gift from Robert Weinberg [17]), empty vector (pBabe-neo, addgene # 1767, a gift from Hartmut Land & Jay Morgenstern & Robert Weinberg [18]), and viral packaging construct pCL-Ampho (a gift from Dr. Hung Nguyen, Center for Cancer Research, National Cancer Institute, Bethesda, MD, USA [19]).

Retroviral particles +/− the hTERT construct were generated by transient co-transfection of pBabe-neo-hTERT or pBabe-neo (5 μg) and pCL-Ampho (2.5 μg) constructs into HEK293T cells grown in collagen coated flasks using the calcium-phosphate method. The following day, the DMEM/F12-5% medium was replaced. Medium containing viral particles was collected 96 h post transfection, passed through a 0.45-μm filter. Subconfluent fibroblast cultures in passage eight were transduced with the viral supernatant supplemented with 8 μg/mL polybrene at serial dilution overnight upon when the medium was replaced. At 90% confluency, the transduced cells underwent antibiotic selection with medium containing 300 μg/mL G418 (Life Technologies) for 9 days until non-transduced control cells showed no signs of survival. The concentration of antibiotic used was determined prior to transduction by testing different concentrations of G418 and choosing the dose of 300 μg/mL G418, which eliminated all cells within 1 week. The transduction efficiency was not more than 15%, in which the majority of cells were transduced by one copy of retroviral particle [20].

### RNA extraction, RT-qPCR, and next generation sequencing

To measure hTERT expression, total RNA was extracted from hTERT-transduced HBFCs, iHBFCs, and empty vector- transduced HBFCs, evHBFCs, in passage 11 according to the manufacturer’s instructions (Sigma, GenElute, RTN70) and the RNA was reverse transcribed to cDNA using the High Capacity RNA-to-cDNA Kit (Applied Biosystems). Real-time quantitative polymerase chain reaction (RT-qPCR) was performed as described [11] using TaqMan Gene Expression Assays (Applied Biosystems) and the TaqMan primers: human telomerase reverse transcriptase (hTERT, Hs00972656_m1), glyceraldehyde-3-phosphate-dehydrogenase (GAPDH, Hs02758991_g1), hypoxanthine phosphoribosyltransferase 1 (HPRT1, Hs99999909_m1), and phosphoglycerate kinase 1 (PGK1, Hs00943178_g1). Gene expression was determined using the formula 1/(2^ΔCT^), in which ΔCT represents the difference between the target and the geometric mean of reference genes. GAPDH, HPRT1, and PGK1 served as reference genes for normalization.

For next generation sequencing, total RNA was extracted using Trizol (Thermo Fischer) and a spin column method according to the manufacturer’s instructions (Zymo Research) from subconfluent duplicate cultures of HBFC^CD105^ and HBFC^CD26^ in passage 9 and from duplicate cultures of passage 24 iHBFC^CD105^ and passage 25 iHBFC^CD26^. RNA sequencing and bioinformatics analysis was performed by the Beijing Genomics Institute (BGI), Hong Kong, as previously described [11]. In brief, sequencing was performed using BGISeq 500 and 13.7 M clean reads were generated for each sample. Mapped clean reads to reference using Bowtie 2 tool [21] were then used to calculate gene expression with the RSEM package [22]. To identify differentially expressed genes (DEGs) between groups, the DESeq2 method was used [23]. A Venn diagram (https://bioinfogp.cnb.csic.es/tools/venny/index.html) was used to depict the overlap of DEGs with a 2-fold difference between fibroblast populations.

For analysis of cluster of differentiation (CD) molecular signature, a comprehensive list of 453 unique CD molecules and their gene names was retrieved from the Uniprot database (https://www.uniprot.org/docs/cdlist) and applied to filter DEGs with a 2-fold difference and FPKM larger than 5. The R software (v3.2.2) was used to plot gene expression values in a heatmap.

### Adipocyte and osteoblast differentiation

To assess adipogenic differentiation, in seven independent tests, iHBFCs in passages 27, 28, 40, 49, and 50 were plated at 40,000 cells/cm^2^ in DMEM/F12-5%. One to two days after plating, the medium was changed to adipogenic inducing medium (MEM-10% with 2.5% horse serum (Sigma Aldrich), 100 nM dexamethasone (Sigma-Aldrich), 500 μM 1-methyl-3-isobutylxanthine (IBMX, Sigma-Aldrich), 1 μM rosiglitazone (BRL49653, Cayman Chemical), and 5 μg/mL insulin (Sigma-Aldrich)) [24]. Controls received MEM-10% medium. The medium was replaced three times per week over 13–25 days on which the cultures were evaluated by Oil Red O staining [25]. Nuclei were counterstained by hematoxylin and photographs were acquired on Leica DM5500B. For osteogenic differentiation, in 5 independent tests, HBFCs in passage 22, 28, 35, 49, and 50 were plated overnight at 20,000 cells/cm^2^ and were then exposed to osteogenic inducing medium (MEM-10% supplemented with 10 mM β-glycerophosphate (Calbiochem), 50 μg/mL L-ascorbic acid (Sigma), 10 nM dexamethasone (Sigma), and 10 nM 1,25-dihydroxy vitamin D3 (LEO Pharma) [26] for 28–32 days with medium change three times a week. Controls received MEM-10% medium. Mineralization was assessed by alizarin red staining [24] and photographs were acquired with Leica Z6 AP0.

### Fluorescence-activated cell sorting and co-cultures

Primary MUC1^high^ luminal epithelial cells (CD271^low^/MUC1^high^) and CD271^high^ myoepithelial cells (CD271^high^/MUC1^low^ or CD271^high^/EpCAM^low^) were isolated from breast tissue biopsies as described [10, 11]. Freshly isolated myoepithelial cells were plated (2500–5000 cells/cm^2^) onto confluent fibroblasts feeder layers of iHBFC^CD105^ and iHBFC^CD26^, respectively. Myoepithelial/fibroblast co-cultures were maintained in a specialized culture medium, Myo medium [11], supplemented with 5% FBS (Myo 5%). In one experiment, cultures were maintained in DMEM/F12-5%, which gave a similar result. Primary myoepithelial cells were also plated on collagen coated plastic in Myo medium and expanded to passage 2 before use in co-cultures with fibroblasts in passage 3 using Myo 5% medium.

To isolate myoepithelial cells from co-cultures, the cell cultures were trypsinized (0.25% trypsin/1 mM EDTA), counted using a Burker-Türk chamber and stained for CD271-APC at 4 °C for 30 min followed by two washes in HEPES/BSA/EDTA buffer. Fixable Viability Stain 780 (1:1000, BD Biosciences) live-dead discriminator was added prior to analysis and sorting on FACS ARIA-II or FACS Fusion (BD Biosciences). FACS data analysis was performed with FACS DIVA and FlowJo software.

In a cross-over test, myoepithelial cells in primary culture were isolated from co-cultures with iHBFC^CD105^ and iHBFC^CD26^, respectively, and from each, 1600 myoepithelial cells/cm^2^ were re-plated onto confluent fibroblast feeders of both iHBFC^CD105^ and iHBFC^CD26^. To account for variance in absolute CD271 levels and for normalization purposes, myoepithelial CD271 levels were divided by the mean background CD271 fluorescence of the co-cultured fibroblasts.

For assessment of epithelial morphogenesis, FACS sorted primary MUC1^high^ luminal cells (6000 cells/cm^2^) were seeded in Myo medium onto confluent feeder layers of iHBFCs and observed for up to 3 weeks using a phase contrast microscope and imaged (Leica DM IL).

In 15 tests using TGF-β signaling inhibition by SB431542 (Axon 1661, Axon Medchem), HBFCs representing four biopsies were allowed to grow to confluence over 7 days and were then treated with 10 μM SB431542 for 3 days before plating of MUC1^high^ luminal cells at day 10 from five biopsies.

In two tests, MUC1^high^ luminal cells from two biopsies were plated onto confluent HBFCs from two biopsies in Myo medium. From days 2–9, the co-cultures were exposed to 10 μM SB431542 or vehicle (DMSO).

### Luminal differentiation

To assess the ability of fibroblasts to direct luminal differentiation capacity of myoepithelial progenitors, fourteen myoepithelial/fibroblast co-cultures (7 pairs of iHBFC^CD105^ and iHBFC^CD26^) representing six different biopsies were used. Specifically, from a pair of co-cultures in DMEM/F12-5% (passage 1) and three co-culture pairs in Myo 5% medium (passages 1, 2 and 3), representing three different biopsies, CD271^high^ myoepithelial cells were isolated by FACS and plated at 1600 cells/cm^2^ for analysis of luminal differentiation. In three other experiments, representing three additional biopsies, primary co-cultures from Myo 5% medium were trypsinized and cells plated without prior FACS sorting into luminal differentiation conditions. For luminal differentiation, conditions were used as described [11], or in some experiments, with similar results, the culture medium was replaced with DMEM/F12 supplemented with 2 mM glutamine, 50 μg/mL gentamycin (Biological Industries), 0.5 μg/mL hydrocortisone (Sigma, H0888), 5 μg/mL insulin (Sigma, I6634), 30 ng/ml epidermal growth factor (recombinant human) (Peprotech), 0.4% (approx. 50 μg/mL) bovine pituitary extract (Gibco, 13-028-014), 20 ng/mL basic fibroblast growth factor (Peprotech), 25 μM Repsox (Sigma, R0158), 4 μg/mL heparin (Sigma), and 20 μL/mL B27 (Life Technologies).

### Immunohistochemistry and immunocytochemistry

Cryostat sections of normal breast tissue biopsies and xenografts as well as cultured cells and cell smears were stained essentially as previously described after fixation in either methanol (M in Table 1) or formaldehyde (F1 in Table 1) or formaldehyde followed by methanol to acetone (F2 in Table 1) and included controls without primary antibody [12, 27, 28]. Blocking was performed for 5 min in 10% goat serum in PBS or Ultra V Block (Lab Vision Corporation TA125-UB). Cells were incubated with primary and secondary antibodies for 60 and 30 min respectively (Table 1). For immunoperoxidase staining, the secondary antibody was UltraVision ONE HRP Polymer (Thermo Fisher, TL-125-PHJ), and for fluorescence, isotype-specific goat anti-mouse IgG AlexaFluor (AF, Life Technologies) secondary antibodies were used. Nuclei of immunoperoxidase- or fluorescence-stained sections and cells were counterstained with hematoxylin or ProLong Gold Antifade reagent with 4,6-diamino-2-phenylindole (DAPI, Life Technologies), respectively.

**Table 1:** List of antibodies and protocols

Antibody	Clone/isotype	Company/catalog no	Peroxidase	Fluorescence	FACS	Fixation
α-SMA	1A4	Sigma/A2547		1:5000		F1
CD105	SN6	Abcam/Ab11414	1:200			F2/M
CD26	202-36	Abcam/Ab3154	1:50			F2/M
CD140b	PR7212	R&D Systems/MAB1263	1:1000–1:2000			F1
CD248	EPR17081	Abcam/ab204914	1:1000–1:2500			F1
K17	E3	DAKO/M7046		1:50		F1/F2/M
K14	LL002	Monosan/MONX10687		1:25–1:50		F1/F2/M
K19	Ba16	GenWay/GWB22664E	1:200			F2/M
K19	Ba16	Abcam/ab20210	1:200	1:50		F2/M
K19	A53-B/A2	Abcam/ab7754		1:100		F/M
CD271	ME20.4	BioLegend/345102		1:25		F1
CD271-APC	ME20.4	Cedarlane/CL10013APC			1:50	
CD326–488	9C4	BioLegend/324210			1:50	
CD326	9C4	BioLegend/324202		1:25		F1
MUC1	115D8	Biogenesis/1510-5025		1:10–1:20	1:50	F2
Vimentin	SP20	Thermo Fisher Scientific/RM-9120	1:200			F1
AF488	IgG1	Life Technologies/A21121		1:500		
AF488	IgG2b	Life Technologies/A21141		1:500	1:500	
AF488	IgG3	Life Technologies/A21151		1:500		
AF568	IgG1	Life Technologies/A21124		1:500		
AF568	IgG2b	Life Technologies/A21144		1:500		
AF647	IgG2a	Life Technologies/A21241		1:500		

Eleven pairs of iHBFC^CD105^ and iHBFC^CD26^ spanning passages 11–50 were stained by immunoperoxidase for CD105 (Abcam, SN6) and CD26 (Abcam, 202–36). Photographs were acquired with Leica DM5500B.

Six to eight micrometers of cryostat sections of three different biopsies were triple-stained by fluorescence for CD271 (BioLegend, ME20.4), α-SMA (Sigma, 1A4), and EpCAM (BioLegend, 9C4) followed by AF488 (IgG1), AF568 (IgG2b), and AF647 (IgG2a). The triple-stainings were evaluated and imaged using confocal microscopy (Zeiss LSM 700).

Six to eight micrometers of cryostat sections of 10 different biopsies were immunoperoxidase-stained for CD140b (PDGFRβ; R&D Systems, PR7212) and CD248 (Abcam, EPR17081), evaluated and imaged (DM5500B).

Xenografts were sectioned (6–8 μm) and co-stained by fluorescence for K19 (Abcam, A53-B/A2) and K14 (Monosan, LL002), followed by incubation with AF568 (IgG2a) and AF488 (IgG3).

MUC1^high^-luminal/fibroblast co-cultures were immunoperoxidase-stained on days 9–12 for Keratin 19 (GenWay or abcam, BA16) and images acquired on Leica Z6 AP0 at 1.25 magnification. The images were analyzed with ImageJ software (v1.52a) in batch mode using a macro previously established [10] counting the number of epithelial structures larger than 0.0026 mm^2^.

For observation of epithelial polarization, 10 pairs of iHBFC^CD105^ and iHBFC^CD26^ in co-culture with luminal epithelial cells from five different biopsies were co-stained on days 9–23 by fluorescence for K19 (Abcam, BA16) and MUC1 (Biogenesis, 115D8) followed by AF488 (IgG2b) and AF568 (IgG1). The co-stainings were evaluated by epi-fluorescence microscopy (Leica DM5500B) and imaged using confocal microscopy (Zeiss LSM 700).

Myoepithelial/fibroblast co-cultures were co-stained for K14 (Monosan, LL002), K17 (Dako, E3) and K19 (Abcam, BA16), followed by AF488 (IgG3), AF568 (IgG2b) and AF568 (IgG1). Images of three co-cultures representing three different biopsies were acquired with Leica DM5500B and K17 intensity measured with image analysis software, ImageJ (1.52a). For this, segmentation was first performed on K14 using the ImageJ functions Multiply, Median, and Make Binary providing the outline of the myoepithelial cells. This segmentation was then applied to corresponding images of K17 in which fluorescence intensity was measured.

Cultures subjected to luminal differentiation conditions were stained for K19 (Abcam, BA16) by immunoperoxidase on days 8–12, evaluated and imaged using Leica DM5500B.

For a quantitative assessment of CD271 as a marker for ductal myoepithelium, cellular smears were prepared from FACS-isolated CD271^high^ versus CD271^low^ myoepithelial cells from four different biopsies. The smeared cells were fixed at room temperature for 10 min in 3.7% paraformaldehyde, washed three times in PBS, and permeabilized in 0.01% Triton X-100 for 10 min followed by three washes in PBS. The fixed smears were blocked by 5 min incubation in Ultra V Block followed by 5 min in 10% goat serum before staining with K17 (Dako, E3) antibody, followed by AF488 (IgG2b) and DAPI. Images of stained smears were acquired with Leica DM5500B and a minimum 100 cells per cell preparation was counted using ImageJ (v1.52a) Cell Counter plugin.

Xenografts were sectioned (6–8 μm) and co-stained by fluorescence for K19 (Abcam, A53-B/A2) and K14 (Monosan, LL002), followed by incubation with AF568 (IgG2a) and AF488 (IgG3) prior to confocal imaging (Zeiss LSM 700).

### In vivo bone formation assay

One million hMSC-TERT (2 implants, 1 mouse) and iHBFC^CD105^ (4 implants, 3 mice) were mixed with 40 mg hydroxyapatite/tricalcium phosphate (HA/TCP) ceramic powder (Zimmer Scandinavia, Albertslund, Denmark), incubated at 37 °C at 5% CO_2_ atmosphere overnight and then implanted subcutaneously in the dorsal side of NOD.CB17-Prkdc^Scid^/J mice (Charles River, France) [29]. Implants were removed after 8 weeks, transferred to 4% neutral buffered formalin for 24 h followed by incubation in formic acid for 3 days. The processed implants were paraffin-embedded, sectioned, and stained as described [30] with human-specific vimentin (Thermo Fisher Scientific, clone SP20) antibody or by hematoxylin-eosin [31].

### In vivo morphogenesis

From primary co-culture with iHBFC^CD105^ or iHBFC^CD26^, approximately 500,000 myoepithelial cells, with or without removal of co-cultured CD271^low^ fibroblasts by FACS, representing two biopsies, were admixed with 125,000 or 500,000 irradiated (~ 20 Gy) iHBFC^CD105^ or iHBFC^CD26^ cells and suspended in cold 1:1 collagen gel to growth factor reduced Matrigel (BD Biosciences) for transplantation. Cells were orthotopically injected into the 4th left and right mammary fat pad of 7–10-week-old female NOD.Cg-*Prkdc*^*SCID*^ *Il2rg*^*tm1sug*^ mice (NOG mice, Taconic) (iHBFC^CD105^: 10 transplants, 5 mice; iHBFC^CD26^: 8 transplants, 4 mice). Mice were supplemented with 0.67 μg/mL 17β-estradiol (Sigma-Aldrich) in the drinking water throughout the experimental period. After 8 weeks, the mice were sacrificed and the mammary glands excised and snap frozen in − 80 °C n-Hexane (Sigma) before mounting for cryostat sectioning.

### Statistics

Statistical analyses and data visualization were performed with a statistical programing language R (version 3.6.3) and its integrated development environment, R studio (version 1.2.5033) and GraphPad Prism (version 8). Estimated *p* values were based on Shapiro-Wilk test for normality, one-way analysis of variance (ANOVA) with Tukey’s test, Kruskal-Wallis rank-sum test, Wilcoxon signed-rank test, or nested *t* test, as indicated.

## Results

### Immortalization of human breast fibroblastic cells (HBFCs)

**Fig. 1:**
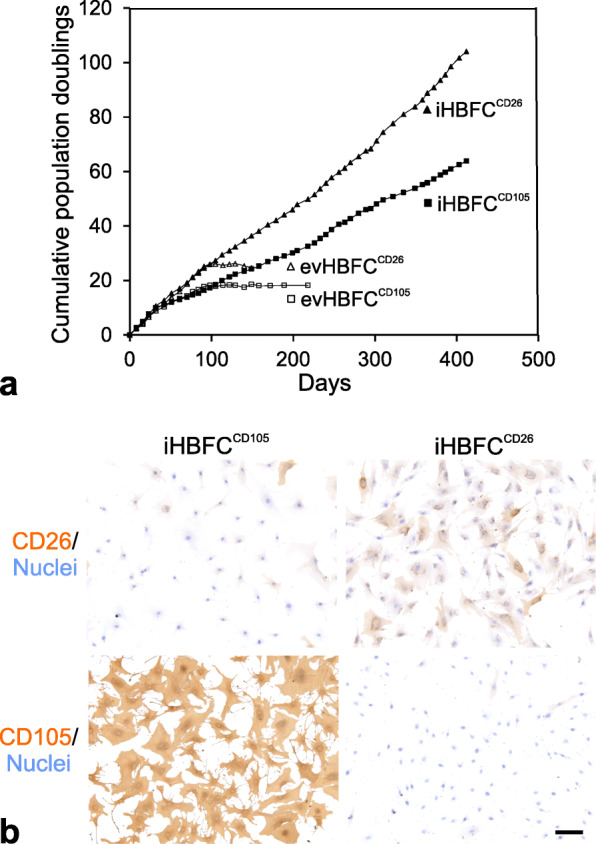
Lineage specific markers are maintained in hTERT immortalized HBFCs. **a** Diagram depicting the cumulative population doublings of CD105^+^ and CD26^+^ HBFCs transduced with empty vector (evHBFC^CD105^; open squares and evHBFC^CD26^; open triangles) or hTERT (iHBFC^CD105^; closed squares and iHBFC^CD26^; closed triangles) and recorded between passages 10 (day zero) and 57 (day 412). Whereas iHBFC^CD105^ and iHBFC^CD26^ continued to proliferate, empty vector controls ceased to expand after around 18 and 24 population doublings, respectively. Also, note that iHBFC^CD26^ have an intrinsic growth advantage irrespective of immortalization. **b** iHBFCs were examined repeatedly for the expression of lineage markers CD105 and CD26 by immunoperoxidase staining (brown), here illustrated for cells in passage 50. Like their primary ancestors, iHBFC^CD105^ are CD105^high^/CD26^low^ (left) and iHBFC^CD26^ are CD105^low^/CD26^high^ (right). Nuclei are counterstained with hematoxylin (blue) (bar = 50 μm)

We previously purified fibroblasts from reduction mammoplasty specimens and sorted them into lobular CD105^high^/CD26^low^ and interlobular CD105^low^/CD26^high^ lineages which could be propagated in culture [10]. Under these conditions, HBFCs senesce after more than 80 days and approximately 16 passages [10]. To generate lines of HBFCs, we here examined whether retroviral transduction with the hTERT gene would render HBFCs immortal. HBFCs in passage eight were infected with retrovirus encoding hTERT together with a neomycin drug resistance marker or an empty vector. Whereas the empty vector cells did not exhibit extended lifespan over what is expected for HBFCs, the hTERT transduced cells generated populations of infected HBFCs with no significant growth arrest and an apparent infinite life span (Fig. 1a and Additional file Fig. 1). Interestingly, the CD105^high^- and CD26^high^-derived cell lines given identical growth conditions, stably exhibited different growth properties (Fig. 1a), and have currently been grown for more than 80 passages. Thus, immortalization was successful, and in the following, we refer to the hTERT transduced breast fibroblasts as iHBFC^CD105^ and iHBFC^CD26^, respectively.

### Differentiation state of iHBFCs

**Fig. 2:**
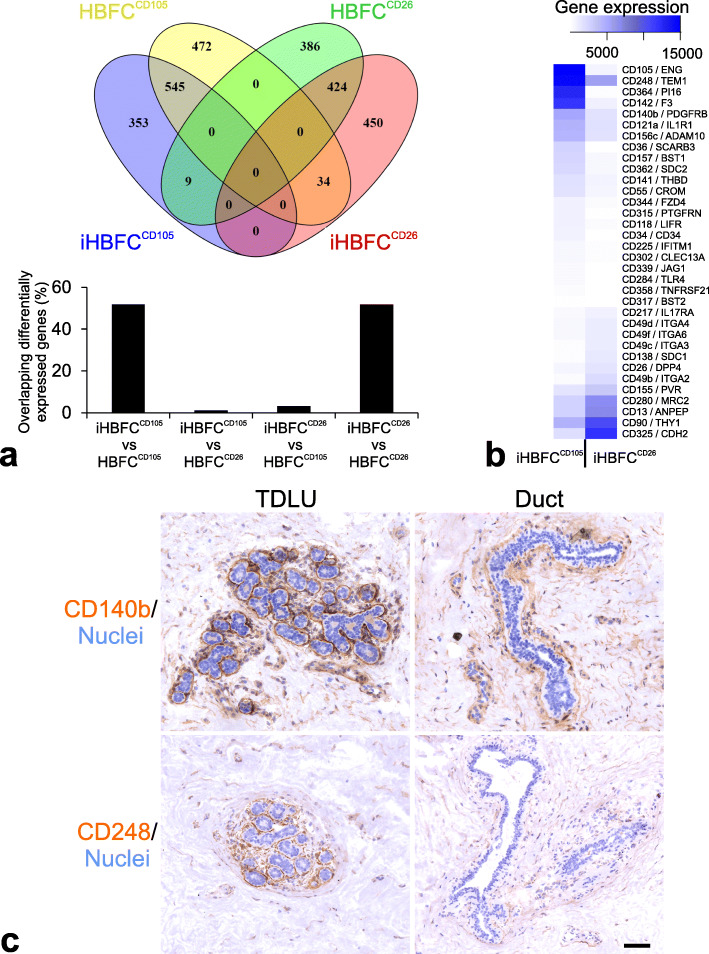
iHBFCs resemble HBFCs by gene expression profiles and CD140b and CD248 are lobular markers in situ. **a** Venn diagram showing the number of differentially expressed genes (DEGs; *p* < 0.05 and fold change ≥ 2) based on genome wide RNA-sequencing of CD105^+^ and CD26^+^ HBFCs and iHBFCs, respectively. Bar diagram shows the percent overlap of DEGs between the cells indicated. **b** Heatmap of expression values of DEGs annotated with a cluster of differentiation (CD) name represented in a for iHBFC^CD105^and iHBFC^CD26^. **c** Cryostat sections of normal breast biopsies stained with peroxidase (brown) for CD140b and CD248 selected based on the CD gene expression profile of iHBFCs. Note the relatively intense staining in TDLUs (left) versus ducts (right). Nuclei are counterstained with hematoxylin (blue) (bar = 100 μm)

To characterize the hTERT immortalized lines, we first examined their staining pattern with CD105 and CD26. As seen in Fig. 1b, iHBFC^CD105^ and iHBFC^CD26^ maintain high expression of CD105 and CD26, respectively (Fig. 1b). In order to further investigate the differences between the two cell lines and in parallel the finite lifespan HBFCs, we next examined the mRNA expression profiles of the iHBFC^CD105^ and iHBFC^CD26^. We found that there were approximately 850–900 transcripts in each population that were > 2-fold differentially expressed compared to the other population and that in general, the iHBFCs remained well differentiated along lobular- and interlobular-fibroblastic pathways, respectively (Fig. 2a). Thus, in contrast to previous attempts to culture and maintain lobular and interlobular breast fibroblast [8, 9] and dermal fibroblast subpopulations [14], the lineages in the present study remain phenotypically distinct in extended culture and upon immortalization.

Since multiple fibroblast subpopulations have been characterized in human dermis based on expression of different combinations of cluster of differentiation (CD) genes [14], we next specifically extracted this information from the mRNA arrays of the iHBFCs (Fig. 2b). The list of 34 genes contained several well-known fibroblast markers, including CD248 (endosialin/TEM1, [32]), CD36 (scavenger receptor class B member 3, SCARB3, [33]), CD34 [34], CD140b (platelet-derived growth factor receptor-beta, PDGFRβ [35];), CD138 (syndecan-1, [36]), CD90 (Thy-1, reviewed in [37]), and CD13 (aminopeptidase N, ANPEP, [8, 38]). Among these, CD90 and CD140b have been defined as pan-fibroblast markers, which are genes expressed at a high level in both papillary and reticular dermal fibroblasts and all cultured fibroblast lines [14]. In the present study, however, the expression levels of these markers appear to distinguish lobular and interlobular iHBFCs, since CD140b is expressed at a higher level in the former, and CD90 is expressed at a higher level in the latter (Fig. 2b). Upon further comparison with human dermis, the most obvious equivalent expressing CD26 is the papillary fibroblast, while CD105 expression concurs with CD36, which is expressed in both lobular breast fibroblasts and lower reticular dermis [39]. Indeed, the iHBFCs serve as a sensible model with relevance to the in vivo setting, which was further illustrated in a series of 10 specimens, where, in addition to CD26 and CD105, two of the identified markers of iHBFCs, CD140b and CD248, recognize the cells in situ which they are supposed to represent (Fig. 2c). This pattern was observed in 8/10 cases. In 2/10 cases, no difference in staining was observed between lobular and interlobular stroma.

Next, we analyzed whether the two cell lineages had also retained critical functional properties in spite of immortality. We have previously shown that CD105^high^ as opposed to CD26^high^ HBFCs in several respects behave like MSCs [10]. Here, we conducted a series of experiments between passage 22 and passage 50 to reveal the potential of the iHBFCs with respect to functional differentiation towards adipocyte and osteoblast lineages. Indeed, the iHBFCs remained discernably stable for the entire culture period with respect to their differentiation potential as demonstrated by accumulation of lipid droplets in adipogenic cultures and formation of mineralized matrix in osteoblastic cultures of iHBFC^CD105^ only (Additional file Fig. 2a and b). Also in this respect, iHBFC^CD105^ show similarity to reticular fibroblasts, which readily undergo adipogenic differentiation [39]. iHBFC^CD105^ do not, however, exhibit the entire differentiation repertoire of MSCs, since they differ from bone marrow-derived MSCs by lack of ability to form bone in vivo (Additional file Fig. 2c). Hence, the iHBFC^CD105^ and iHBFC^CD26^ retain critical properties of primary cells and of their putative cells of origin and share lineage relationships with fibroblasts from other tissues.

### Fibroblast cell type and impact on breast epithelial progenitors

**Fig. 3:**
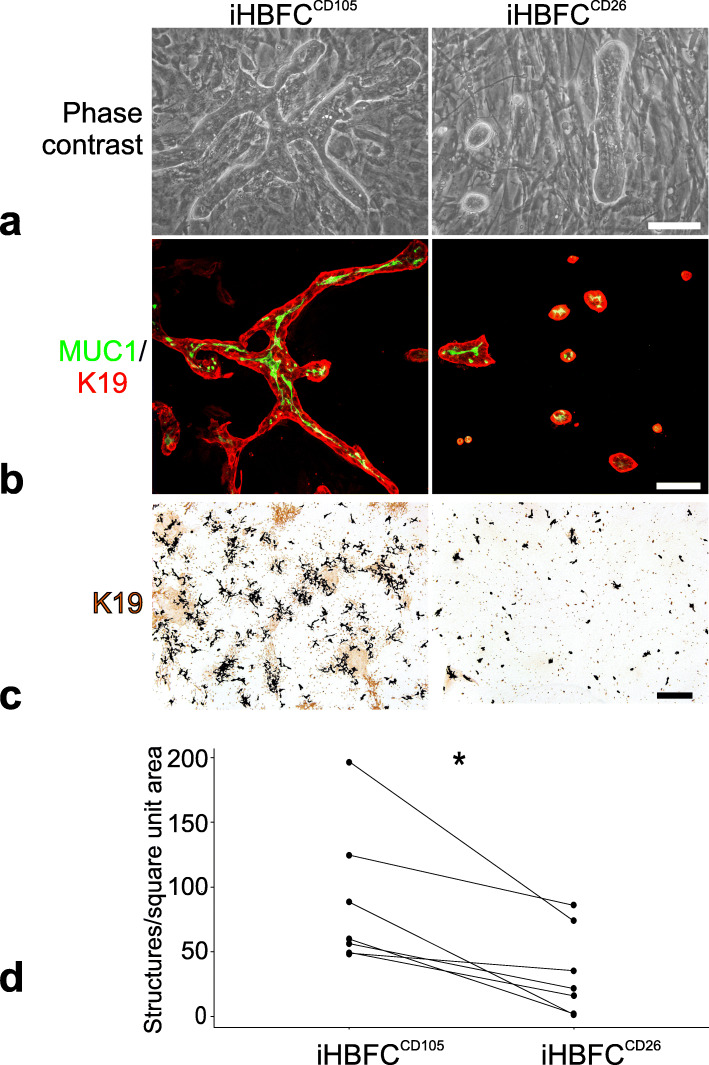
iHBFC^CD105^ support luminal epithelial growth and TDLU-like branching morphogenesis. Comparison of the capacity of iHBFC^CD105^ and iHBFC^CD26^ to induce human breast epithelial morphogenesis. **a** Phase contrast micrographs of luminal breast epithelial cells co-cultured for 16 days on passage 40 iHBFC^CD105^ (left) or iHBFC^CD26^ (right) (bar = 100 μm). Only iHBFC^CD105^ facilitate elaborate TDLU-like branching morphogenesis. **b** Double immunofluorescence staining of luminal epithelial/iHBFC co-cultures with K19 (red) and MUC1 (green; bar = 100 μm). Note the staining of correctly polarized MUC1 in K19^+^ structures in both co-cultures. **c** Illustration of difference in induced branching morphogenesis by iHBFC^CD105^ and iHBFC^CD26^, respectively, by low magnification imaging and segmentation in ImageJ of branching morphogenesis in luminal epithelial/iHBFC co-cultures stained by peroxidase for K19 (brown). Segmented images show epithelial structures projected in black pixels (bar = 1000 μm). **d** Dot plot depicting the inductive capacity of seven pairs of iHBFC^CD105^ (left) and iHBFC^CD26^ (right) measured as the number of luminal epithelial structures per square unit area using luminal epithelial cells from five different biopsies. Consistently, iHBFC^CD105^ have higher inductive capacity (asterisk indicates significance at *p* < 0.05 by Wilcoxon signed-rank test)

**Fig. 4:**
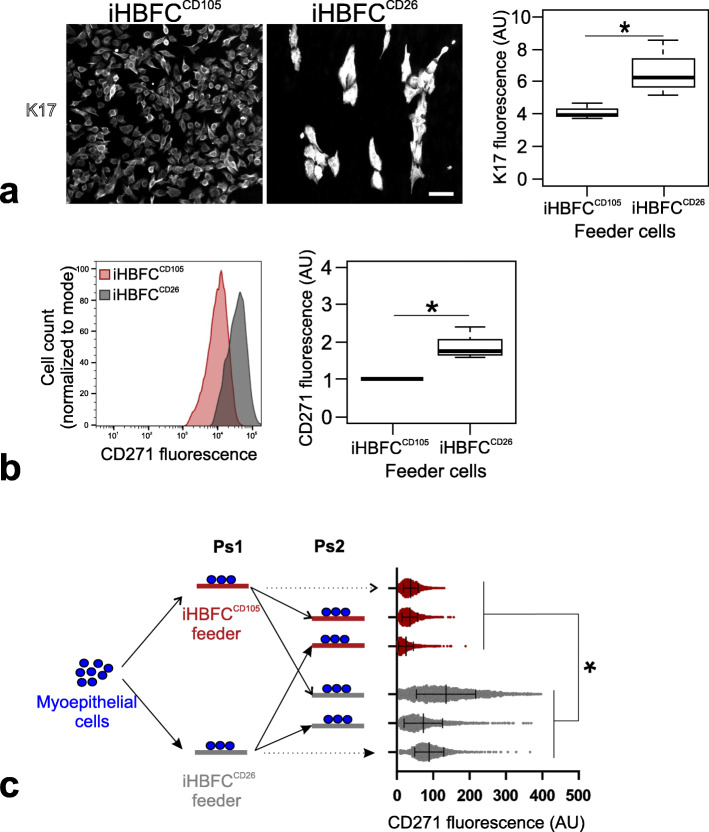
iHBFC^CD26^ convey a ductal-like differentiation of myoepithelial cells. **a** Images showing FACS sorted CD271^high^/MUC1^low^ breast primary myoepithelial cells in co-culture with iHBFC^CD105^ (left) and iHBFC^CD26^ (right), fluorescently labeled for K17 (white) and K14 (not shown) by immunocytochemistry. K14 staining was used as a guide in image analysis to identify K14^+^ myoepithelial cells prior to measuring myoepithelial K17 staining intensity. Box plot shows interquartile range and median of K17 mean fluorescence in arbitrary units (AU) of three biopsies (whiskers indicate upper and lower quartiles; asterisk indicates significance at *p* < 0.05 by Kruskal-Wallis rank-sum test). **b** Primary myoepithelial/fibroblast co-cultures (iHBFC^CD105^ (red), iHBFC^CD26^ (gray)), were single cell suspended and stained for CD271 before analysis by FACS. Histogram shows cell count normalized to mode versus myoepithelial CD271 staining intensity in arbitrary units (AU) of a single biopsy (left) and box plot shows the interquartile range and median of the mean of CD271 fluorescence intensity relative to iHBFC^CD105^ in arbitrary units of three biopsies (right; whiskers indicate upper and lower quartile, asterisk indicates significance at *p* < 0.05 by Kruskal-Wallis rank-sum test). **c** Schematic showing the experimental outline (left): primary CD271^+^ myoepithelial cells are plated onto confluent fibroblast feeders (passage 1 co-culture, Ps1), from which myoepithelial cells are isolated and then re-plated onto new fibroblast feeders (passage 2 co-culture, Ps2). Dot plot (right) shows normalized myoepithelial CD271 fluorescence in arbitrary units (AU) with mean values and standard deviations indicated by vertical bars as measured by FACS of 2250 cells in passage 1 and 2 co-cultures grouped according to feeder (iHBFC^CD105^ (red) or iHBFC^CD26^ (gray)). Note that the myoepithelial phenotype shifts as a consequence of a switch between fibroblasts (asterisk indicates significance at *p* < 0.05 by nested *t* test)

With a reproducible source of lobular- and interlobular-like HBFCs in hand, we assessed their impact on the neighboring breast epithelium. Firstly, we looked at the luminal epithelial compartment characterized by a high cellular turnover in vivo [12]. Here, we took advantage of a heterotypic co-culture assay designed for measuring branching morphogenesis [10, 40]. As seen in Fig. 3, the readout from this assay was an unequivocal high level of branching morphogenesis supported by iHBFC^CD105^. This difference between iHBFC^CD105^ and iHBFC^CD26^ in inductive capacity was robust throughout the entire culture period from passages 14 to 47 and was independent of source of epithelial cells (Fig. 3). Secondly, we looked at the myoepithelial compartment, which is believed to contain the apical-most progenitors in the human breast hierarchy [41–43]. Here, we took advantage of the fact that ductal and lobular myoepithelial cells in situ differ in both their marker expression and their differentiation potential [11]. The question remains as to whether these properties to some extent rely on topographical conditions such as those determined by the adjacent fibroblasts. To address this, we isolated the entire complement of myoepithelial cells from three different biopsies by a CD271 FACS protocol. These myoepithelial cells were plated directly on either iHBFC^CD26^ or iHBFC^CD105^ and cultured for 1 week followed by staining for keratin K17 (Fig. 4a) and CD271 (Fig. 4b). Notably, the readout for ductal-like myoepithelial differentiation was based on both high CD271 and high keratin K17 since these co-segregated in FACS and stainings (Additional file Fig. 3). Interestingly, ductal-like, high expression of both CD271 and K17 entirely relied on co-culture with iHBFC^CD26^. That fibroblasts indeed influence epithelial differentiation was further substantiated by passaging the cells to a second passage with switching of the feeders. Now, those myoepithelial cells that were initially ductal-like in phenotype with high CD271 expression became lobular-like with reduced CD271 expression and vice versa (Fig. 4c). This indicates that the myoepithelial phenotype is regulated by surrounding fibroblasts.

**Fig. 5:**
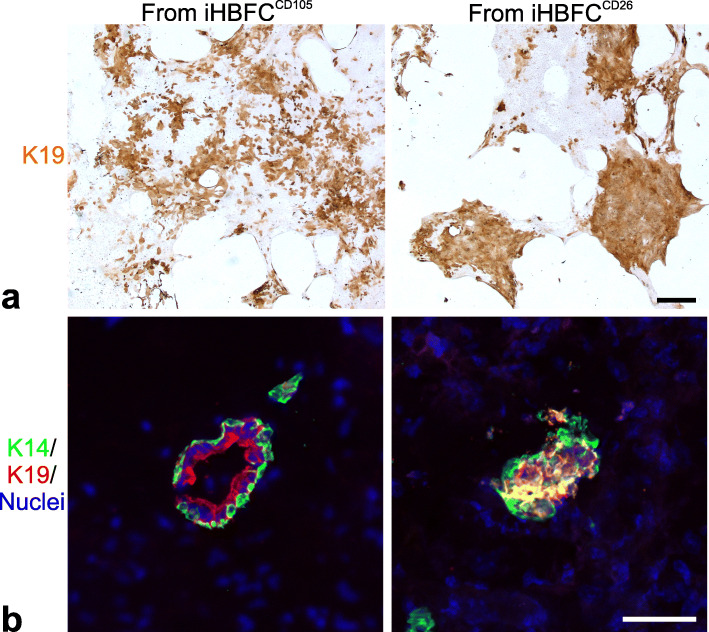
The luminal differentiation repertoire of myoepithelial progenitors is directed by interaction with specialized fibroblasts. **a** Comparison of capacity of fibroblasts to direct epithelial progenitor capacity. Myoepithelial cells co-cultured with iHBFC^CD105^ or iHBFC^CD26^ were passaged and subjected to luminal differentiation conditions at clonal density and peroxidase stained for K19. While the induced K19 appeared mainly scattered when derived from iHBFC^CD105^ co-culture (left), additional rather homogenous islets presented from iHBFC^CD26^ co-cultures (right). The distinct phenotypes were observed in five out of seven tests with absence of homogeneous islets from iHBFC^CD26^ in two tests (bar = 500 μm). **b** Representative multicolor confocal images (K19, red; K14, green; nuclei, blue) of cryostat sections of xenografted NOG mice 8 weeks after orthotopic injection of myoepithelial cells from primary co-culture with iHBFC^CD105^ or iHBFC^CD26^. Bilayered epithelial structures were obtained in 6/10 and 5/8 injections from iHBFC^CD105^ and iHBFC^CD26^, respectively, although at limited numbers, down to a few per transplant. Whereas iHBFC^CD105^ co-culture derived myoepithelial cells readily differentiated into luminal K14^−/low^/K19^+^ cells, co-culture with iHBFC^CD26^ resulted mainly in K14^+^/K19^+^ luminal cells (bar = 50 μm)

Whether this also applies to the next level of differentiation potential of myoepithelial cells, i.e., generation of luminal cells, was examined by measuring the pattern of induced luminal keratin K19 in myoepithelial progenitors under differentiating conditions. Whereas lobular-like luminal differentiation is characterized by emergence of scattered heterogeneous islets of K19-positive luminal cells, ductal-like luminal differentiation entails homogeneous islets reminiscent of their differentiation in vivo [11]. Accordingly, myoepithelial cells primed by co-culture with either iHBFC^CD105^ or iHBFC^CD26^ were plated at clonal density under identical luminal differentiation conditions without fibroblast feeders [11]. Based on experiments with 6 different biopsies we found that priming with either iHBFC^CD105^ or iHBFC^CD26^ impacted on the following luminal differentiation potential corresponding to preferentially scattered or homogeneous keratin K19 staining, respectively (Fig. 5a). This observation was further validated in vivo. Myoepithelial cells primed in co-culture with either iHBFC^CD105^ or iHBFC^CD26^ orthotopically injected into NOG mice resulted in bilayered epithelial structures from both origins in 6/10 and 5/8 transplants, respectively. However, while iHBFC^CD105^ co-culture- derived myoepithelial cells gave rise to K14^−/low^/K19^+^ cells, iHBFC^CD26^ co-culture-derived myoepithelial cells gave rise to K14^+^/K19^+^ luminal cells (Fig. 5b). Taken together, these results imply that fibroblasts influence epithelial progenitors and that lobular fibroblasts support the development of a more mature luminal phenotype characteristic of TDLU.

### Interruption of a TGF-β signaling cascade in HBFC^CD105^ and control of epithelial progenitors

**Fig. 6:**
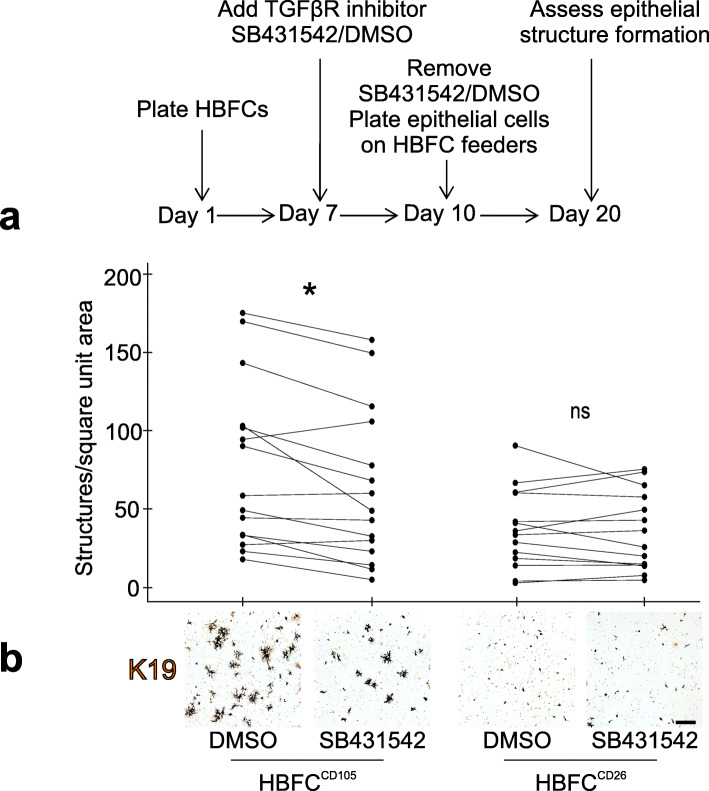
HBFC^CD105^ TGF-β signaling supports parenchymal morphogenesis. **a** Overview of experimental design in which HBFCs are plated and exposed to 10 μM SB431542 or vehicle (DMSO) from day 7 to day 10 at which SB431542 or vehicle are removed and primary CD271^low^/MUC1^high^ luminal breast epithelial cells are added and co-cultured for 10 days prior to assessment of epithelial structure formation. **b** 15 tests representing recombinations of four fibroblast biopsies and five epithelial biopsies are presented in a paired dot plot and a representative set of micrographs, showing a significant reduction in epithelial structure formation per square unit area in response to SB431542 versus vehicle in HBFC^CD105^ co-cultures only (asterisk indicates significance at *p* < 0.05 by Wilcoxon signed-rank test, ns = not significant) (bar = 1000 μm)

Since lobular fibroblasts exhibit a TGF-β signaling signature [10] and CD105 is a co-receptor for TGF-β (reviewed in [44]), we speculated whether the TGF-β signaling pathway plays a role in the crosstalk between fibroblasts and epithelial progenitors. To explore this, we used the quantitative morphogenesis assay described above and initially incubated luminal epithelial-fibroblast co-cultures directly with the small molecule TGF-β signaling inhibitor, SB431542, previously shown by others to impinge on CD105 signaling [45]. Indeed, in 2 out of 2 tests, the number of epithelial structures in HBFC^CD105^, but not in HBFC^CD26^ co-cultures, was reduced by treatment with SB431542 (Additional file Fig. 4). To exclusively target the fibroblasts, we then incubated confluent fibroblast feeders with SB431542 for 3 days prior to plating of the luminal cells on top. Disruption of TGF-β signaling significantly reduced epithelial structure formation in HBFC^CD105^ co-cultures, but not in HBFC^CD26^ co-cultures (Fig. 6). This result suggests that intact TGF-β signaling in lobular fibroblasts is instrumental in modulating parenchymal cells.

## Discussion

A number of contextual signals have been implicated in the maintenance of tissue homeostasis in the human breast some of which originating from neighboring fibroblasts and impacting on stem cell behavior (reviewed in [46]). Also, it has long been suspected that fibroblasts exhibit functional specialization according to their anatomical location [9, 47, 48], but it still remains an open question which cells identify the stromal microenvironment, and how they are specified for the production of proliferation and differentiation cues [46]. Our research in adult breast tissue has revealed the existence of two distinct lineages—a lobular and an interlobular which remain inherently functionally distinct [10]. Here, using hTERT expression vectors, we have been able to generate two populations of cells that reside stably in the lobular-like and interlobular-like states, respectively, as defined by a number of properties including the CD105 and CD26 expression. The resulting fibroblast cell lines are faithful to their identity corresponding to their anatomical site of origin, and specifically, lobular-like fibroblasts, relying on a TGF-β signaling pathway, govern epithelial morphogenesis and differentiation typical of the TDLU.

The above observations leave several questions unanswered about the role of fibroblasts in the human breast. We have previously shown that lobular-derived and ductal-derived epithelial cells maintain their distinct properties either in the absence of fibroblasts, which is in three-dimensional culture within a reconstituted basement membrane, or in co-culture on mouse-derived fibroblasts (3T3-cells) suggesting that epithelial cells are not submitted to modulation by microenvironmental cues [11, 12]. In the present study, however, we show that early myoepithelial progenitors are susceptible to cues from lobular- and interlobular-like fibroblasts in terms of luminal differentiation repertoire. Thus, if primed on lobular-like fibroblasts, luminal differentiation is more elaborate, which is reminiscent of the luminal lineage in TDLUs in situ. On the other hand, if myoepithelial progenitors are primed with interlobular-like fibroblasts, the luminal differentiation is limited to K14 and K19 double-positive progenitors both in culture and in vivo. It is possible that human breast epithelial progenitors for appropriate interaction with the surrounding stroma rely on species-specific crosstalk. This notion is supported by an experimental paradigm described more than a decade ago, when it was shown that normal morphogenesis and differentiation of human breast epithelial cells transplanted into mice required co-implantation with human fibroblasts [49]. Our present findings extend this observation to include plasticity of prospectively isolated human breast progenitors as determined by positional information from resident fibroblasts.

Lobular-like human breast fibroblasts generated either by prospective FACS isolation from primary tissue or through hTERT immortalization exhibit a strong expression of CD105. A number of studies have indicated that CD105 modulates TGF-β signaling through ALK5 and responds to bone morphogenic proteins (BMPs) (reviewed in [50]). BMPs also play an important role in maintenance and specification of human breast stem cells [51]. Consistent with this, we found that inhibition of the TGF-β signaling cascade by pre-incubation with SB431542 specifically in the lobular-like fibroblasts led to attenuated interaction with epithelial progenitors in the subsequent co-culture experiment. Although still not completely elucidated, it appears that such TGF-β dependent epithelial-stromal interaction is crucial also for cancer development. While TGF-β1 converts the majority of normal breast fibroblasts to alpha-smooth muscle actin-positive myofibroblasts [15], disrupted TGF-β-signaling attenuates CAF-induced cancer cell growth [52].

We show here that while lobular-like fibroblasts in many respects are similar to human bone marrow-derived MSCs, they fail in an ultimate in vivo test gauging for bone formation. Thus, as far as the human breast is concerned, we can now distinguish resident fibroblasts from bona fide MSCs. This is important because the latter has been implicated in reactive stroma formation such as that occurring in cancer. Thus, it has been speculated that MSCs are recruited to the breast as a source of myofibroblasts or CAFs responsible for important aspects of tumor cell-stroma interaction including promotion of metastasis ([53–55], reviewed in [56]). With the in vivo bone formation assay employed here, the question of a “third” immigrant mesenchymal lineage in breast pathology can be addressed also in a human context. Such investigations are ongoing in our laboratory.

The fibroblast heterogeneity described herein is likely to be in operation in a wider variety of tissues and organs. In the present study, we demonstrate by genome wide gene expression profiling that lobular-like and interlobular-like fibroblasts differ by entire lineage programs with characteristics and functions in common with previously reported papillary and reticular fibroblasts, respectively, in mice and humans [1, 14, 39]. In this regard, it is interesting that CD26^−^ fibroblasts in mice segregate into mature CD26^+^ papillary fibroblasts [1] and that in both mice and humans such fibroblasts are responsible for ECM production and in turn fibrosis [57–59]. We propose that the CD26^+^ interlobular-like fibroblasts are responsible for the dense fibrous tissue of the breast and further responsible for the differences in breast density between individuals – a known risk factor for development of breast cancer. This would concur with the observation that another marker, CD36, expressed by lobular fibroblasts, is repressed in high density breast stroma [33]. CD105^+^ lobular-like fibroblasts on the other hand have properties in common with bone marrow-derived MSCs and reticular fibroblast progenitors, which participate in wound healing and myofibroblast generation [1]. Previous results from our laboratory have shown that lobular fibroblasts readily generate α-smooth muscle actin-positive myofibroblasts [10] and that interlobular fibroblasts exhibit an immune related gene expression profile [10]. Whether the breast cancer repertoire of CAFs is a caricature and maybe even a reminiscence of the normal stromal cell heterogeneity remains an open question. Interestingly, however, recent single-cell RNA sequencing of breast carcinomas has resolved stromal cell diversity to include both myofibroblastic and inflammatory CAFs and not least perivascular cells [60, 61]. While this concurs with our early studies, which suggested diverse cellular origins of CAFs, including resident fibroblasts and perivascular cells [62, 63], our present findings suggest that lineage heterogeneity within the resident fibroblast compartment adds to the complexity. If indeed this is the case and myofibroblast and inflammatory classification operate among both CAFs and normal resident fibroblasts, it is tempting to speculate on lineage interrelationships and how these may be taken advantage of in a clinical setting [64]. The cell lines established in the present study may prove valuable in determining such lineage relationships. It is also a possibility that phenotypic and functional CAF heterogeneity reflects plasticity in a broader sense and in general may be governed more or less by the tumor genotype as suggested in mouse models of pancreatic cancer (reviewed in [65]). In this context, the cell lines may serve to decipher whether a specific tumor genotype instructs development of a particular stromal response independent of recipient initial stromal cell type.

For these reasons, it is likely that both lobular and interlobular-like fibroblasts play important albeit different roles in normal breast as well as in breast cancer.

## Conclusions

Collectively, our study shows that we have established two physiologically relevant, phenotypically distinct human breast fibroblast cell lines, which exhibit specialized functions in maintenance of region-specific characteristics and regulation of neighboring epithelial cells. In the longer perspective, the present developments may provide a basis for the experimentation in cell-based assays to elucidate the earliest events in human breast cancer evolution.

## Supplementary information

**Additional file 1: Figure S1.** iHBFCs express hTERT. Bar graph depicting the relative hTERT expression in arbitrary units (AU) assessed by RT-qPCR in triplicate normalized to the geometric mean of reference genes GAPDH, HPRT1 and PGK1. hTERT expression was detected in cells transduced with hTERT (iHBFC^CD105^ and iHBFC^CD26^) but not in cells transduced with the empty vector (evHBFC^CD105^ and evHBFC^CD26^). Error bars represent mean ± SD.

**Additional file 2: Figure S2.** iHBFC^CD105^ are MSC-like but lack in vivo osteogenic differentiation potential. (a,b) Comparison of the potential of iHBFC^CD105^ and iHBFC^CD26^ cells to undergo adipogenic and osteogenic differentiation. (a) Micrographs of cells exposed to adipogenic inducing conditions followed by staining with Oil Red O and hematoxylin. Prominent perinuclear accumulation of lipid droplets is seen in iHBFC^CD105^ cells only (left). The stainings are representative of five independent experiments with cells in up to passage 50, (bar = 50 μm). (b) Quantification of matrix mineralization upon exposure to standard medium (−) or osteogenic inducing medium (OIM; +) followed by staining with alizarin red. Significant matrix mineralization is restricted to iHBFC^CD105^ (left; asterisk indicates *p* < 0.05 tested by one-way Anova with Tukey’s honest significance test). Matrix mineralization was repeatedly tested positive in iHBFC^CD105^ in up to passage 50. Bars represent the mean of three independent experiments ± SD. AU: arbitrary units. (c) iHBFC^CD105^ and hMSC-TERT cells were mixed with hydroxyapatite/tricalcium and implanted subcutaneously into immunodeficient mice. Implants were removed after eight weeks, processed for staining by human specific vimentin (top row, brown) and hematoxylin/eosin (H&E, bottom row). Positive human-specific vimentin staining indicates presence of the implanted cells. White dotted outlines indicate normal lamellar bone formed by hMSC-TERT, which is absent in iHBFC^CD105^ transplants, (bar = 50 μm).

**Additional file 3: Figure S3.** Myoepithelial CD271 expression is higher in ducts than in TDLUs**.** (**a**) Representative images of normal breast cryostat sections stained by immunofluorescence for α-smooth muscle actin (α-SMA, green, top panel) and CD271 (green, bottom panel) and nuclei counterstained with DAPI (blue) (*n* = 3 biopsies). Positive staining for α-SMA reveals myoepithelial cells in both TDLUs (left) and ducts (right). In three out of three biopsies, the myoepithelium in ducts exhibited more intense staining for CD271 relative to the myoepithelium in TDLUs. (**b**) Representative FACS diagram of a trypsinized breast organoid preparation stained by CD271 and CD326 from which CD271^high^ and CD271^low^ myoepithelial cells were isolated (gates indicated by circles), smeared and stained by immunofluorescence for K17 (green) and nuclei (blue). (**c**) Histogram showing enrichment in percent of K17^+^ cells among CD271^high^ versus CD271^low^ myoepithelial cells in four out of four biopsies, (bar = 50 μm).

**Additional file 4: Figure S4.** Disruption of TGF-β signaling decreases epithelial morphogenesis in HBFC^CD105^ co-cultures. Primary CD271^low^/MUC1^high^ luminal epithelial cells from two different biopsies were plated onto confluent fibroblasts feeders and the resulting co-cultures were exposed to 10 μM SB431542 or vehicle (DMSO) from day 2 after epithelial plating. At day 9 the number of structures per square unit area was assessed as illustrated in Fig. 3. While the number of epithelial structures on HBFC^CD105^ is reduced by SB431542, the capacity of HBFC^CD26^ to influence epithelial morphogenesis apparently is not affected by the TGF-β signaling inhibitor.

## Data Availability

The RNA sequencing dataset comparing lobular and interlobular fibroblasts generated and analyzed during the current study is available in the Gene Expression Omnibus (GEO) repository, [GEO accession number GSE153646, https://www.ncbi.nlm.nih.gov/geo/query/acc.cgi?acc=GSE153646].

## Abbreviations

CAF: Carcinoma-associated fibroblast
CD: Cluster of differentiation
FACS: Fluorescence-activated cell sorting
hTERT: Human telomerase reverse transcriptase
iHBFC: Immortalized human breast fibroblastic cell
MSC: Mesenchymal stem cell
MUC1: Mucin 1
TDLU: Terminal duct lobular unit
TGF-β: Transforming growth factor-beta
