# Supplementary figures and images for: Fibroblasts direct differentiation of human breast epithelial progenitors
Source: Breast Cancer Res. 2020 Sep 29;22:102. doi: 10.1186/s13058-020-01344-0 (PMC7526135; doi:10.1186/s13058-020-01344-0)

## Additional file Figure 1: iHBFCs express hTERT

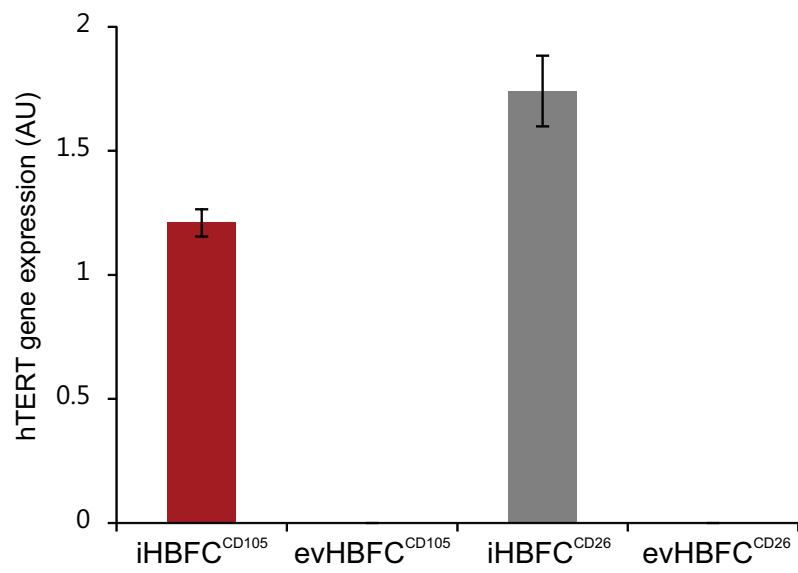

Supplement: Supplementary file 1 — Additional file 1: Figure S1. iHBFCs express hTERT. Bar graph depicting the relative hTERT expression in arbitrary units (AU) assessed by RT-qPCR in triplicate normalized to the geometric mean of reference genes GAPDH, HPRT1 and PGK1. hTERT expression was detected in cells transduced with hTERT (iHBFCCD105 and iHBFCCD26) but not in cells transduced with the empty vector (evHBFCCD105 and evHBFCCD26). Error bars represent mean ± SD. [file 13058_2020_1344_MOESM1_ESM.pdf]

**Additional file Figure 3: Myoepithelial CD271 expression is higher in ducts than in TDLUs**

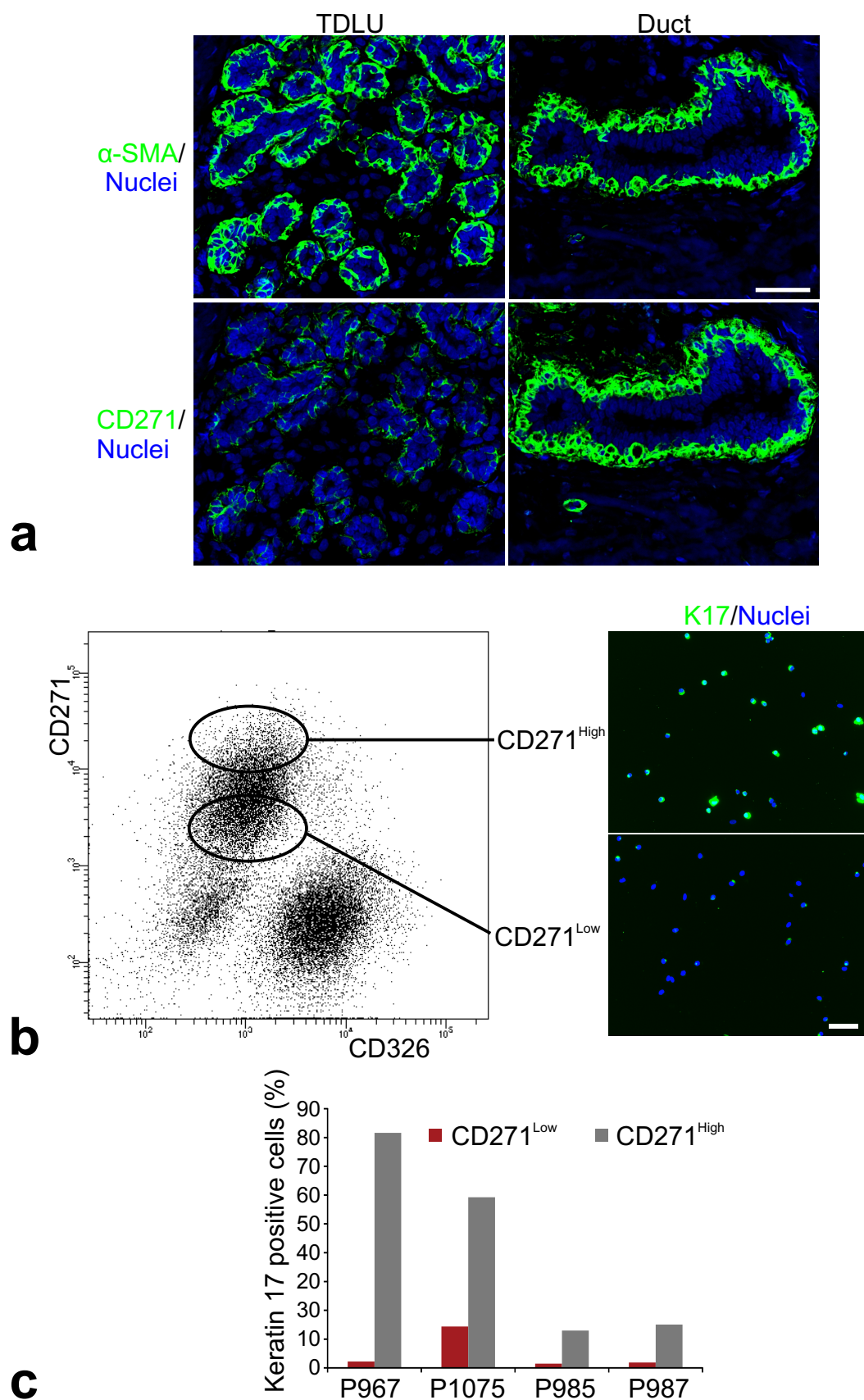

Supplement: Supplementary file 3 — Additional file 3: Figure S3. Myoepithelial CD271 expression is higher in ducts than in TDLUs. (a) Representative images of normal breast cryostat sections stained by immunofluorescence for α-smooth muscle actin (α-SMA, green, top panel) and CD271 (green, bottom panel) and nuclei counterstained with DAPI (blue) (n = 3 biopsies). Positive staining for α-SMA reveals myoepithelial cells in both TDLUs (left) and ducts (right). In three out of three biopsies, the myoepithelium in ducts exhibited more intense staining for CD271 relative to the myoepithelium in TDLUs. (b) Representative FACS diagram of a trypsinized breast organoid preparation stained by CD271 and CD326 from which CD271high and CD271low myoepithelial cells were isolated (gates indicated by circles), smeared and stained by immunofluorescence for K17 (green) and nuclei (blue). (c) Histogram showing enrichment in percent of K17+ cells among CD271high versus CD271low myoepithelial cells in four out of four biopsies, (bar = 50 μm). [file 13058_2020_1344_MOESM3_ESM.pdf]
