# Supplementary material for: Fibroblasts direct differentiation of human breast epithelial progenitors
Source: Breast Cancer Res. 2020 Sep 29;22:102. doi: 10.1186/s13058-020-01344-0 (PMC7526135; doi:10.1186/s13058-020-01344-0)

Additional file Figure 2: iHBFC^{CD105} are MSC-like but lack in vivo osteogenic differentiation potential

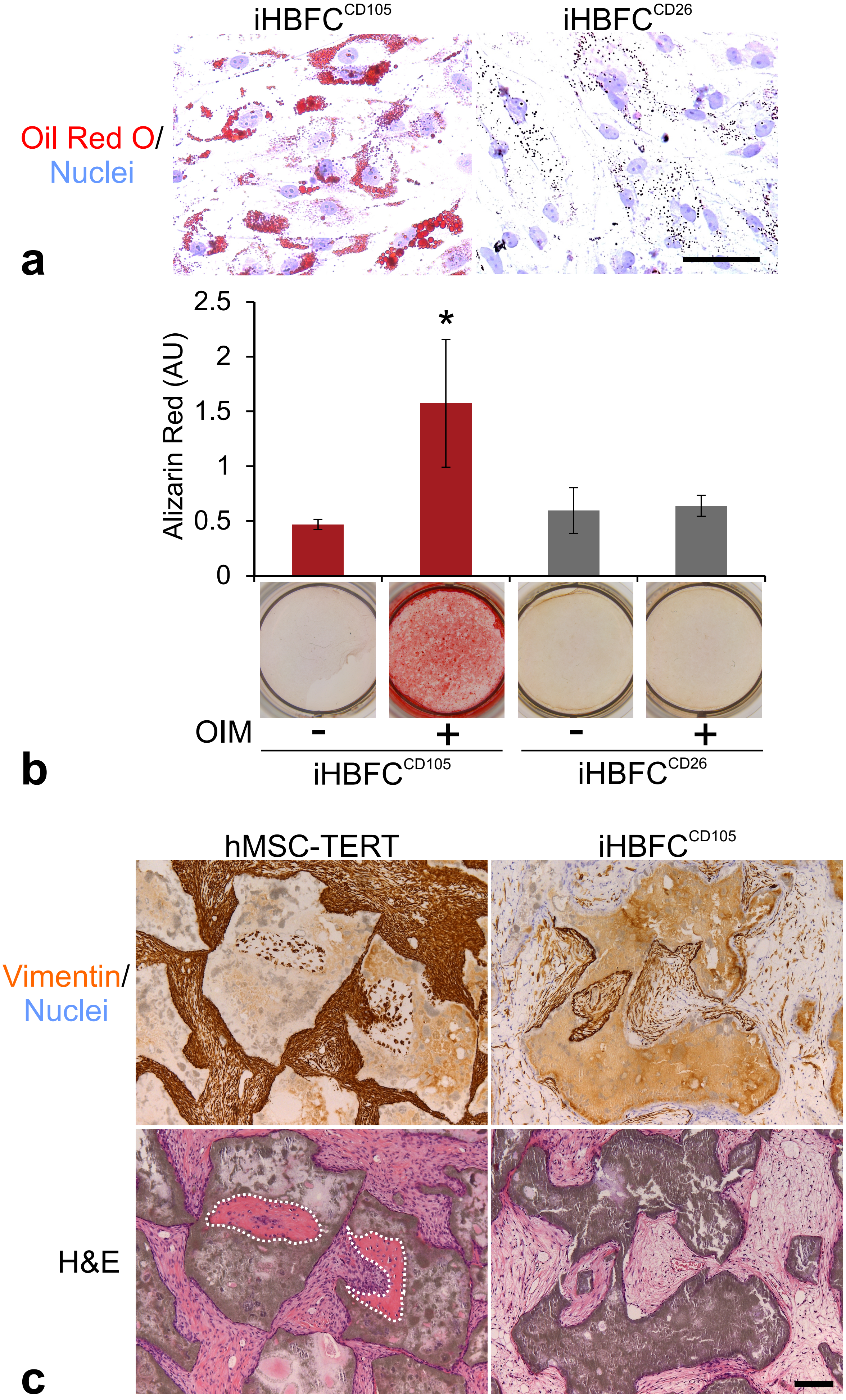

Supplement: Supplementary file 2 — Additional file 2: Figure S2. iHBFCCD105 are MSC-like but lack in vivo osteogenic differentiation potential. (a,b) Comparison of the potential of iHBFCCD105 and iHBFCCD26 cells to undergo adipogenic and osteogenic differentiation. (a) Micrographs of cells exposed to adipogenic inducing conditions followed by staining with Oil Red O and hematoxylin. Prominent perinuclear accumulation of lipid droplets is seen in iHBFCCD105 cells only (left). The stainings are representative of five independent experiments with cells in up to passage 50, (bar = 50 μm). (b) Quantification of matrix mineralization upon exposure to standard medium (−) or osteogenic inducing medium (OIM; +) followed by staining with alizarin red. Significant matrix mineralization is restricted to iHBFCCD105 (left; asterisk indicates p < 0.05 tested by one-way Anova with Tukey’s honest significance test). Matrix mineralization was repeatedly tested positive in iHBFCCD105 in up to passage 50. Bars represent the mean of three independent experiments ± SD. AU: arbitrary units. (c) iHBFCCD105 and hMSC-TERT cells were mixed with hydroxyapatite/tricalcium and implanted subcutaneously into immunodeficient mice. Implants were removed after eight weeks, processed for staining by human specific vimentin (top row, brown) and hematoxylin/eosin (H&E, bottom row). Positive human-specific vimentin staining indicates presence of the implanted cells. White dotted outlines indicate normal lamellar bone formed by hMSC-TERT, which is absent in iHBFCCD105 transplants, (bar = 50 μm). [file 13058_2020_1344_MOESM2_ESM.pdf]
