# Supplementary material for: Fibroblasts direct differentiation of human breast epithelial progenitors
Source: Breast Cancer Res. 2020 Sep 29;22:102. doi: 10.1186/s13058-020-01344-0 (PMC7526135; doi:10.1186/s13058-020-01344-0)

Additional file Figure 4: Disruption of TGF-β signaling decreases epithelial morphogenesis in $HBFC^{CD105}$ co-cultures

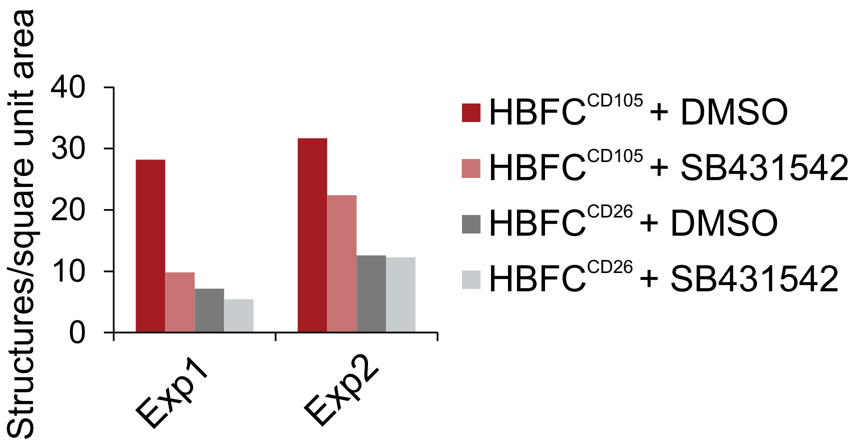

Supplement: Supplementary file 4 — Additional file 4: Figure S4. Disruption of TGF-β signaling decreases epithelial morphogenesis in HBFCCD105 co-cultures. Primary CD271low/MUC1high luminal epithelial cells from two different biopsies were plated onto confluent fibroblasts feeders and the resulting co-cultures were exposed to 10 μM SB431542 or vehicle (DMSO) from day 2 after epithelial plating. At day 9 the number of structures per square unit area was assessed as illustrated in Fig. 3. While the number of epithelial structures on HBFCCD105 is reduced by SB431542, the capacity of HBFCCD26 to influence epithelial morphogenesis apparently is not affected by the TGF-β signaling inhibitor. [file 13058_2020_1344_MOESM4_ESM.pdf]
